# Intrinsic Effect of Pyridine-N-Position on Structural Properties of Cu-Based Low-Dimensional Coordination Frameworks

**DOI:** 10.3390/ijms21176171

**Published:** 2020-08-26

**Authors:** Anna Walczak, Gracjan Kurpik, Artur R. Stefankiewicz

**Affiliations:** 1 Faculty of Chemistry, Adam Mickiewicz University, Uniwersytetu Poznańskiego 8, 61-614 Poznań, Poland; anna.jenczak@amu.edu.pl (A.W.); grakur@amu.edu.pl (G.K.); 2 Center for Advanced Technologies, Uniwersytetu Poznańskiego 10, 61-614 Poznań, Poland

**Keywords:** pyridyl-*β*-diketones, ambidentate ligands, copper (II) complexes, metallosupramolecular architectures, coordination polymers

## Abstract

Metal-organic assemblies have received significant attention for catalytic and other applications, including gas and energy storage, due to their porosity and thermal/chemical stability. Here, we report the synthesis and physicochemical characterization of three metallosupramolecular assemblies consisting of isomeric ambidentate pyridyl-*β*-diketonate ligands L1–L3 and Cu(II) metal ions. It has been demonstrated that the topology and dimensionality of generated supramolecular aggregates depend on the location of the pyridine nitrogen donor atom in L1–L3. This is seen in characterization of two distinct 2D polymeric assemblies, i.e., [Cu(L1)_2_]_n_ and [Cu(L2)_2_]_n_, in which both β-diketonate and pyridine groups are coordinated to the Cu(II) center, as well as in characterization of the mononuclear 1D complex Cu(L3)_2_, in which the central atom is bound only by two β-diketonate units.

## 1. Introduction

Coordination-driven self-assembly processes harness the directionality and reversibility of metal–ligand interactions to allow formation of supramolecular architectures with strictly defined shape and topology [1,2,3,4,5,6]. The spontaneous but controlled generation of well-defined multimetallic architectures is seen in the formation of one-, two- and three-dimensional coordination polymers (CPs) with numerous applications in, e.g., magnetism, molecular separations, dye degradation and catalysis [7,8,9]. The great advantage of CPs over other types of supramolecular materials is their facile chemical modification by changing, e.g., the position/type of donor atoms or metal ion coordination geometry. By combining appropriate metal cations with carefully selected organic linkers (ligands), the structural properties and topology of CPs can be easily modulated and adapted to a desired function, e.g., in catalysis or sorption/separation processes [10,11].

Coordination complexes containing ambidentate ligands that are capable of binding metal-ion centres in more than one way through different donor-atom combinations provide an efficient strategy in the construction of functional coordination assemblies [12,13].Of the many structurally distinct ambidentate ligands, pyridyl-β-diketonates have been widely employed in the generation of different coordination systems from simple mononuclear complexes, [14] through cages [15] and macrocycles [16] to larger architectures (e.g., polymers (CPs) and metal-organic frameworks) [15,17,18,19,20,21,22,23] Importantly, coordination structures based on ambidentate ligands have shown real potential in many applications including catalysis, [24,25,26] bioinorganic modelling [27,28] and molecule magnetism [29,30,31] and in the design of molecular machines [32]. More recently, ambidentate ligands have been successfully utilized with metal ions such as Pd(II), Pt(II) and Cu(I) to provide a series of metallosupramolecular complexes with different levels of dynamicity and high catalytic activity in important processes such as the Suzuki–Miyaura, alkene hydrosilylation and Ullmann reactions [24,25,26].

**Figure 1:**
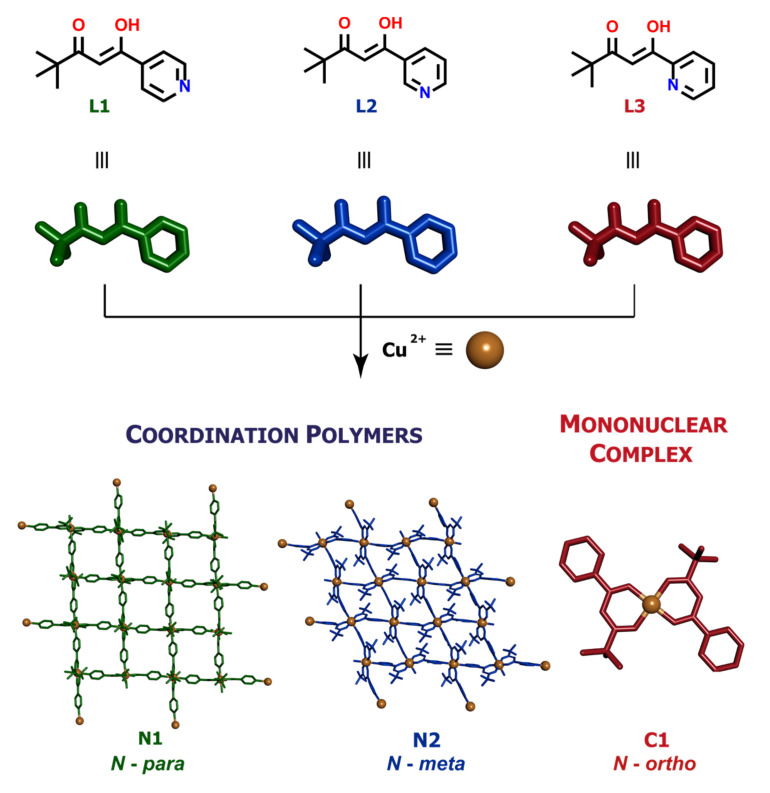
Synthetic routes for the Cu(II) metallosupramolecular architectures based on the pyridyl-β-diketonate ligands.

The isomeric ligands L1, L2 and L3 (Figure 1) have a number of possible coordination modes, even on a single metal centre. As neutral species, all could act as *O,O*-chelates through the β-diketone unit (in either its true diketone or keto-enol form) or as simple *N*-donors through the pyridine ring, with only L3 having the additional possibility of acting as an *N,O*-chelate. As neutral but zwitterionic species resulting from proton transfer from O to N, all three could act exclusively as *O,O*-chelates. In their anionic forms resulting from deprotonation of the β-diketone unit, the same possibilities arise as that for the neutral ligand, although it would be anticipated that the negatively charged diketonate site would now be much more strongly favoured over the neutral *N*-site. In no case is it possible for all donor atoms to be bound to one metal ion so that such ligands must have the capacity to act as bridging species in coordination polymers and metal-organic frameworks, the dimensionality of such species being determined by the stereochemistry of the metal ion involved. Thus, L1–L3 together with an appropriately selected metal ion were expected to be suitable for the synthesis of topologically distinct low-dimensional (1D and 2D) coordination frameworks. Indeed, bridging by the anionic form of ligands related to L1 and L2 by replacement of the *tert*-*butyl* group by methyl or phenyl has been observed in their Cu(II) coordination polymers [33,34]. In addition, other supramolecular interactions encoded in the structure of the ligands were expected to play an important role in determining and stabilizing such types of extended metal-ion assemblies. Despite the fact that low-dimensional coordination assemblies present more flexible frameworks than 3D polymers, little attention has so far been paid to, e.g., the adsorption properties of this type of metal-organic systems. Thus, by combination of Cu(II) metal centres with the three isomeric pyridyl-β-diketonates ligands L1–L3, we anticipated that the metallosupramolecular assemblies obtained (N1–N2 and C1) would differ not only with respect to their structure and topology but also, as a result of that, in physicochemical properties as well. The present work describes our efforts to explore this hypothesis. 

## 2. Results and Discussion

### 2.1. Synthesis and Analysis

**Figure 2:**
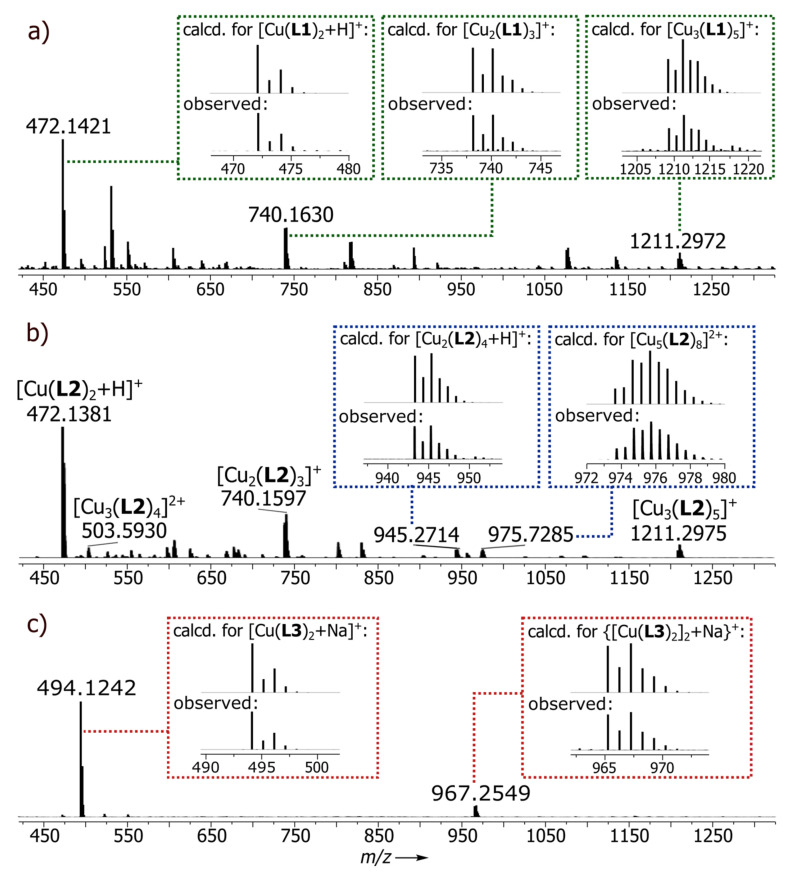
ESI-Q-TOF-HRMS spectra of Cu(II) metal-organic assemblies (**a**) N1, (**b**) N2 and (**c**) C1.

The ligands were prepared by Claisen condensation, following a modified literature procedure and involving the reaction between the methyl esters of isonicotinic (for L1), nicotinic (for L2) or picolinic (for L3) acids and pinacolone in the presence of a strong base [24,35]. Depending on the position of the nitrogen atom in the pyridine ring, structurally distinct assemblies were obtained: two coordination polymers N1 (*para*-*N*) and N2 (*meta*-*N*) and a monomeric complex C1 (*ortho*-*N*). All complexes were obtained by the reaction between the appropriate ligand and Cu(II) salt in 2:1 ratio. Although the reported complexes are neutral species, the reactant counterion was of great importance. The best yields were obtained when acetate or nitrate were employed. With other salts such as halides or sulphates, highly insoluble material immediately precipitated from the reaction mixture, preventing further analysis. Optimal procedures with different ligands varied slightly. Best yields in C1 synthesis were obtained when copper acetate was reacted with L3 at room temperature in THF/MeOH (tetrahydrofuran/methanol) (1:1, *v/v*). That procedure proved to be inefficient in preparing N1 and N2, where better yields were obtained when the reaction was carried out in the presence of a stronger base (Na_2_CO_3_) and at a slightly elevated temperature (40 °C). In each case, green crystals were formed by slow evaporation of the reaction mixture. Crystallization of polymers N1 and N2 took a long time (around 6 weeks), giving crystals of good quality. The solid complexes were characterized by elemental analysis, single crystal X-ray diffraction and XRD powder diffraction. High-resolution ESI-MS spectrometry also demonstrated the presence of the expected fragmentary constituents of each (Figure 2, Figures S4, S7 and S10).

### 2.2. X-ray Structure Determinations

#### 2.2.1. Ligand [HL3][NO_3_] 

**Figure 3:**
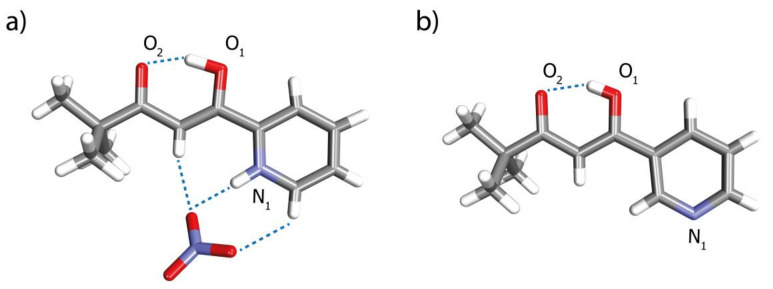
X-ray structures of (**a**) ionic L3 and (**b**) neutral L2 reported previously [24].

During one attempted preparation of complex C1, crystals of the uncoordinated ligand L3 in the form of nitrate salt were obtained. The crystal structure established protonation of the pyridine nitrogen atom and H-bonding interactions between methine, two aromatic protons and the nitrate counterion (Figure 3a). The protonated ligand in its crystal adopts its enol form similarly to the previously described neutral version of L2 (Figure 3b) [24]. This tautomer is stabilized by a strong resonance-assisted intramolecular hydrogen bond [36] involving O1-H⋯O2 (1.618 Å).

#### 2.2.2. Network N1

**Figure 4:**
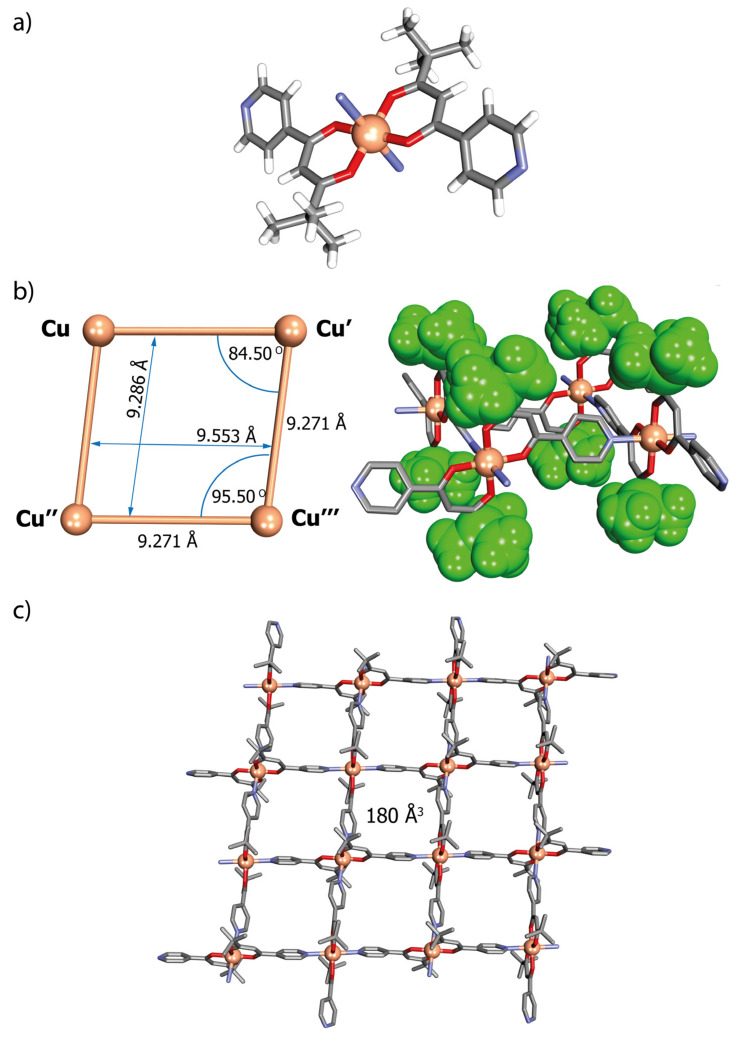
The crystal structure of [Cu(L1)_2_]_n_ (N1) showing (**a**) one of the [Cu(L1)_2_] units coordinated to two pyridine nitrogen atoms from neighbouring metalloligands; (**b**) one of the Cu_4_ rhombic-grid units with cavity dimensions; and (**c**) the two-dimensional single sheet structure adopted by N1 parallel to the (101) crystallographic plane: H-atoms have been omitted for clarity.

The structure of [Cu(L1)_2_]_n_ in solid state was established by a single crystal X-ray determination as being built up from sheets of a two-dimensional rhombic grid-like coordination polymer lying parallel to the (101) crystallographic plane, analogous to previously reported examples [33,34]. All Cu atoms are equivalent, and each is six coordinates with distorted octahedral geometry (*C2_h_* local symmetry), being bound within an O_4_ plane from two *trans*-oriented β-diketonate groups (Cu–O 1.954(1) and 1.958(1) Å) with two axially located nitrogen atoms from pyridyl groups (Cu–N 2.396(1) Å, Figure 4a). Each almost square, grid-like Cu_4_ unit, edge length Cu···Cu 9.271 Å, defines with its ligands a rhomboidal cavity with dimensions of 9.286 × 9.553 Å and a calculated interior volume of 180 Å^3^ (Figure 4b,c and Figure S13). This cavity contains no solvent molecules, as confirmed by thermogravimetric (TGA) analysis (see below). The *trans* arrangement of the two diketonate ligands on Cu means that the *tert*-*butyl* substituents at each Cu centre lie above and below the mean plane of the polymer, thus producing a lipophilic face on each side of the sheet.

**Figure 5:**
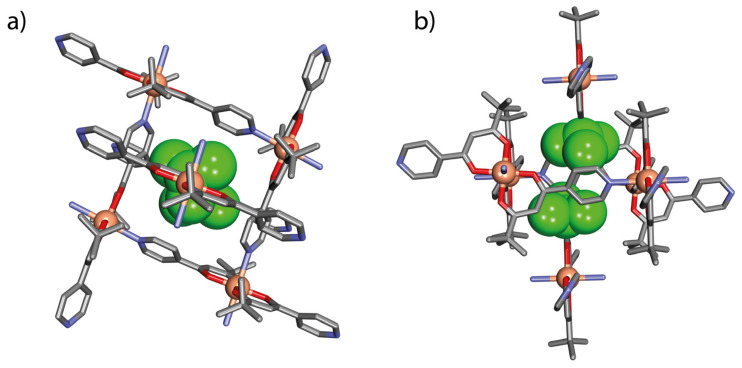
Self-inclusion between adjacent coordination layers of C1: (**a**) top view parallel to the (101) crystallographic plane and (**b**) side view parallel to the (5-4-3) crystallographic plane. H-atoms have been omitted for clarity.

Packing of the sheets of polymers is such that the cavities in each layer are capped by the *tert*-butyl groups projecting from adjacent layers (Figure 5). This leads to dispersion interactions between the methyl groups and the pyridine units forming the wall of the cavity. These intermolecular weak forces may produce flexible parts in the porous network so that the system can exhibit certain dynamicity in the solid state, depending on the external perturbations. The layer-to-layer self-inclusion may somewhat hinder access of the guest molecules inside the pores of the N1 structure but does not exclude application of complexes with such characteristics in gas sorption [37].

#### 2.2.3. Network N2

**Figure 6:**
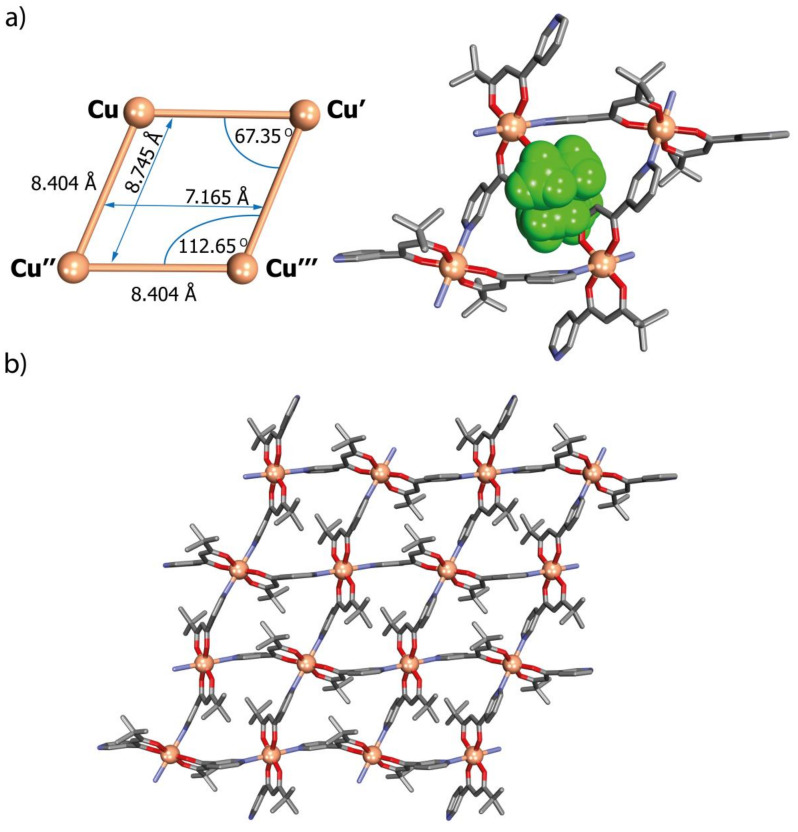
(**a**) Aspects of the geometry of Cu_4_ rhombic-grid units (left) with a view of the cavity occupied by the *tert*-butyl groups (right) and (**b**) view of the 2D network of network N2 parallel to the (10-1) crystallographic plane: H-atoms have been omitted for clarity. Disorder of the *tert*-butyl groups is not shown.

Crystals of the polymer [Cu(L2)_2_]_n_ suitable for crystallographic measurements were obtained by slow evaporation of a THF solution. The polymer crystallizes as shiny green rhombic blocks in the monoclinic space group P2_1_/n. As in N1, all Cu atoms are equivalent and have, as a result again of binding to both diketonate and pyridine donors, the same *C2_h_*-symmetric *trans*-N_2_O_4_ coordination geometry, with very similar bond lengths (Cu-O, 1.949(1) and 1.955(1) Å; Cu-N, 2.409(2) Å). The change in position of the nitrogen donor atom from *para* (N1) to *meta* (N2), although not affecting the coordination mode around the central atom, causes several significant changes in the structure. Although a 2D polymer forming sheets parallel, here, to (10-1) is again present, the Cu_4_ units which can be regarded as having been fused to form the polymeric sheet are much further from square (Figure 6), with a shorter side length (8.40(1) Å), and the sheet itself is much more compact. Thus, the rhomboidal cavity of the Cu_4_ unit is both strongly tilted and smaller (6.600 × 8.573 Å), and in contrast to N1, the *tert*-butyl groups are directed into the cavity, with the external faces of the sheet being made up of both aliphatic and aromatic (pyridine) CH units. Despite similarity to a system reported by Domasevitch, [33] entirely different packing is observed in the case of the system described by Gloe and Lindoy, showing disordered structure, in which two types of five-coordinated Cu(II) occur [34].

#### 2.2.4. Complex C1

**Figure 7:**
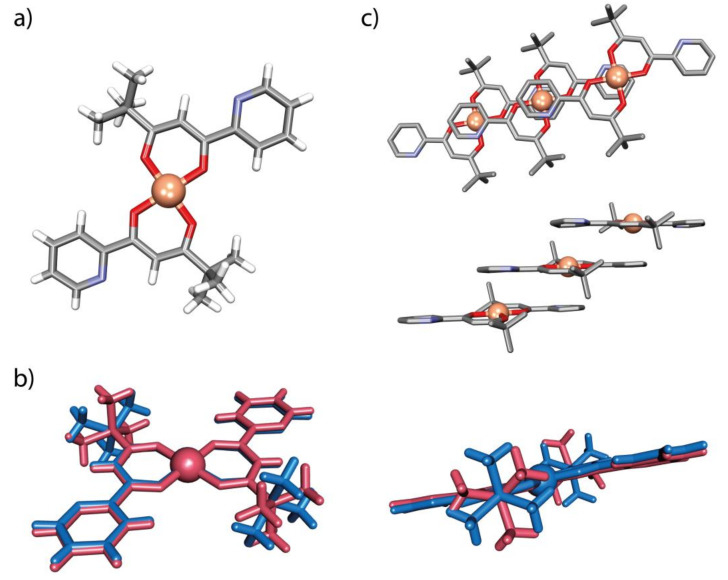
(**a**) The molecular species within the crystal structure of Cu(L3)_2_ (tC1); (**b**) superposition of the molecules of tC1 (red) and mC1 (blue); and (**c**) part of the 1D stacks (top parallel to the (-3-1-2) crystallographic plane and side views parallel to the (-12-1) crystallographic plane) of parallel molecules found in tC1: H-atoms have been omitted for clarity.

While *N,O* chelation would seem to be a possible mode of coordination for L3, it is clearly not favoured over *O,O* chelation (of the diketonate form), an interaction which appears to inhibit any *N*-coordination and thus polymerization, as shown by the presence of molecular bis(diketonate) species in the crystal structures of the isolated complex. In contrast to the previously reported Cu complex with phenyl substituted linker, [34] C1 crystallizes in two distinct polymorphic forms, triclinic P-1 (tC1) and monoclinic P2_1_/n (mC1), in both of which the complex has a distorted square planar geometry around the copper centre with two anionic *O,O’*-chelates from *β*-diketonate moieties coordinated in a *trans* planar fashion (Figure 7a) [38] There are only very minor differences between the polymorphs (Figure 7b). The Cu–O distances are 1.904–1.907 Å and 1.914–1.917 Å for tC1 and mC1, respectively. In both polymorphs, the diketonate and attached pyridine units are close to coplanar, indicating a significant degree of conjugation. In the monoclinic form, the slipped stacks of complex units which lie in columns parallel to the *b* axis (Figure 7) are linked by weak CH···N interactions involving the pyridine-*N* and its adjacent CH, while in the triclinic form, these interactions, now between columns lying parallel to *a*, appear to be much weaker. In both slipped stacks, the interplanar distances are quite short (3.370 Å for tC1 and 3.217 Å for mC1), indicating significant dispersion interactions. The noncovalent ligation in the apical position in C1 may impose some degree of flexibility in the crystal lattice [39].

### 2.3. Powder X-ray Diffraction (PXRD) Analyses

**Figure 8:**
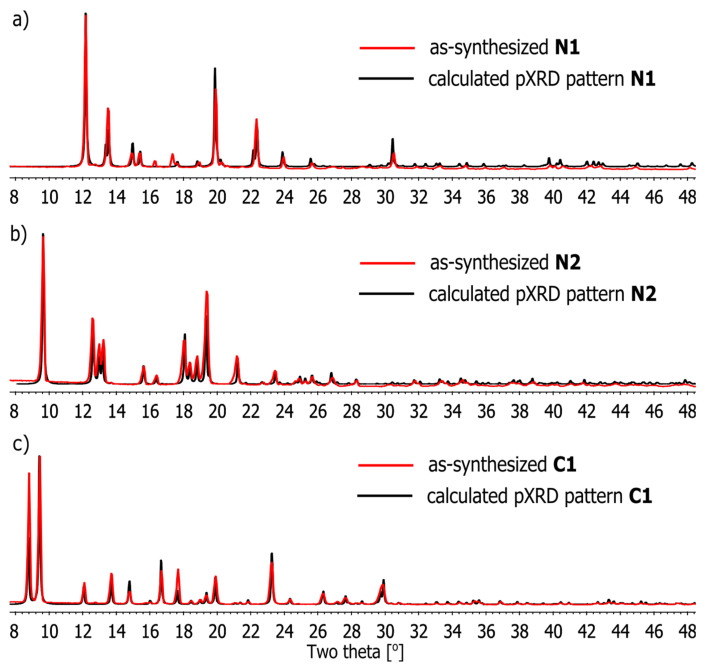
Comparison of powder X-ray diffraction patterns of compounds (**a**) N1, (**b**) N2 and (**c**) C1, as-synthesized (red) and calculated (black) from single-crystal X-ray data of N1–N2 and C1.

The phase purities of as-synthesized samples of coordination compounds N1–N2 and C1 in solid state (Figure 8) were characterized by Powder X-ray Diffraction (PXRD) at 25 °C. The experimental PXRD patterns correspond well with those simulated from the single-crystal data, indicating the bulk samples to be a single phase. The as-synthesized C1 PXRD pattern is well-indexed to the calculation of the triclinic phase. In addition, the PXRD profiles indicate that the solids retain their crystallinity on being crushed. The minor difference in reflection intensities observed between the simulated and experimental patterns may be attributed to variation in the preferred orientation of the powder samples during collection of the experimental PXRD data.

### 2.4. SEM Analysis 

**Figure 9:**
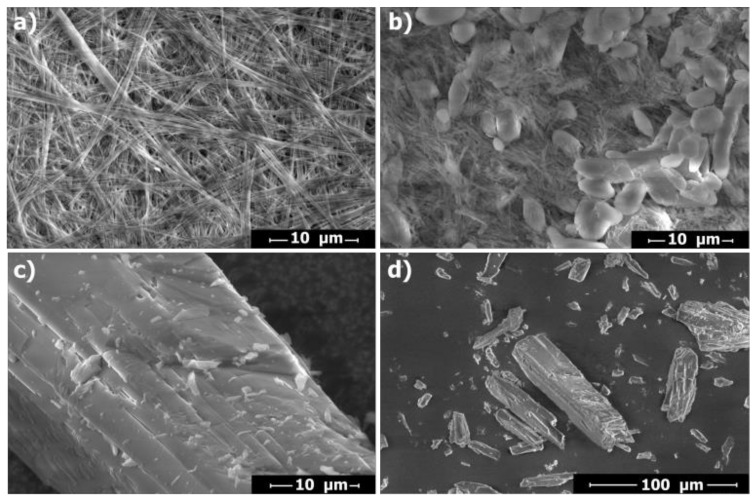
Scanning electron microscopy (SEM) images of complexes (**a**) N1, (**b**) N2 and (**c**,**d**) triclinic form C1.

The solid coordination polymers (N1 and N2) were investigated by scanning electron microscopy (SEM) to get visual insights into their morphologies. The images were recorded with an accelerating voltage of 20 kV and magnification up to ×30,000 using an LFD (large field detector) in solid state. Initially, the powdered samples of the appropriate complex were applied to a Si/SiO_2_ substrate and dried under vacuum. The morphologies of coordination polymers N1 and N2 were found to be quite distinct, though the complexes differ only in the position of the nitrogen atom in the pyridine ring. The SEM images of N1 (Figure 9a and Figure S6) indicate the formation of fibre-shaped morphology, with an estimated average length of up to 20 µm. The fibres intertwine in three dimensions, giving a dense-packed network. The SEM image of N2 exhibits diverse polymeric morphology, with the average length of the fibres decreased by 5 times (approx. 4 µm, Figure 9b and Figure S9). The shrinkage in fibre length of N2 occurs probably due to the formation of a more densely packed coordination network, which is consistent with the data obtained from the X-ray studies. The SEM analysis divulges that the synthesized coordination assemblies remained in polymeric form and confirms the significant impact of the polymers’ crystal packing on the morphology of the nanostructure formed. For comparison, SEM images were also recorded for the C1 complex (Figure 9c,d and Figure S12). In contrast to the two previously discussed assemblies, C1 appear as single microcrystals rather than polymeric networks, approx. 20–100 µm with a triclinic crystalline phase. 

### 2.5. Framework Thermal Stability

**Figure 10:**
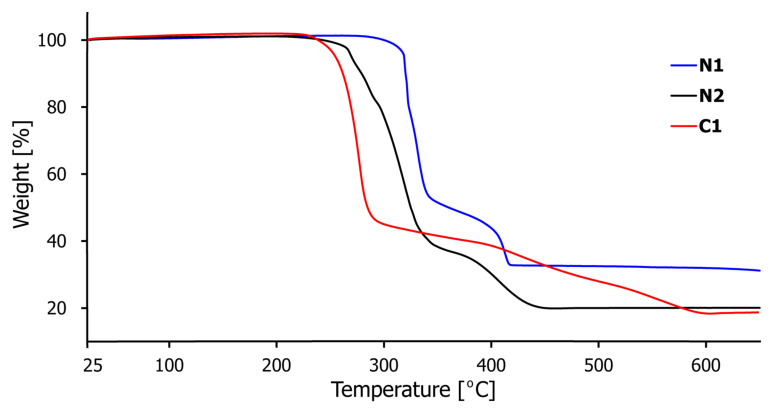
The thermogravimetric analysis (TGA) curves for coordination polymers N1–N2 and complex C1.

Thermogravimetric analysis (TGA) was conducted to study the thermal stability of compounds N1–N2 and C1 (Figure 10). The experiments were performed on crystalline samples (approx. 5 mg) in the temperature range 25–650 °C under N_2_ atmosphere with a heating rate of 10 °C/min. The TGA curves of polymers N1 and N2 have similar profiles and show high stability of these materials with no weight loss of up to 300 and 250 °C, respectively, after which the framework structure begins to collapse (Figure 10, Figures S5 and S8, blue and black curves, respectively). Two-stage decomposition via gradual removal of organic components of compounds N1 and N2 occurs from 300 and 250 °C to 420 and 440 °C, respectively, leading to the formation of copper oxides as the residue (Cu_2_O for N1 calcd. 30.31% and found 30.02% and CuO for N2 calcd. 16.85% and found 18.90%). The TGA pattern of C1 is slightly different (Figure 10 and Figure S11, red curve), which results from the dimeric nature of this complex compared to polymeric N1 and N2. The crystal plateaus steadily until 230 °C, after which a stepwise decomposition of the organic ligands occurs (from 230 to 590 °C), finally giving predominantly a CuO residue (calcd. 14.62% and found 16.34%). 

### 2.6. Gas Sorption Studies

CPs have been proposed as promising gas adsorbents due to their high stability and well-defined porous structures. While these materials are intensively focused on frameworks consisting of polydentate linkers, neutral systems based on ambidentate ligands are much less explored [40,41,42,43,44]. This prompts us to evaluate gas sorption properties of N1–N2 and C1 despite relatively low porosity of the complexes as indicated by X-ray analysis (Table S1). The adsorption/desorption isotherms for N_2_ were measured for degassed frameworks of N1–N2 and C1 at 77.35 K (Figure S14). All three complexes presented very low sorption values in the pressure range of 0–800 mmHg (Table S2), consistent with the fact that these materials have no obvious pores. The values of Brunauer–Emmett–Teller isotherm (BET) surface areas (1.9–4.3 m^2^g^−1^) obtained for all three complexes confirmed their nonporous nature, and despite their promising 2D topology, potential applications in this area would require considerable structural modifications in the ligand structure. 

## 3. Materials and Methods 

### 3.1. General Methods

All chemicals and solvents were obtained from commercial sources and used as received. THF was dried by distillation with benzophenone/sodium prior to use. The ^1^H NMR spectra were acquired on a Bruker Fourier 300 spectrometer equipped with a ^1^H 5 mm probe and referenced to the solvent residual peaks. All spectra were acquired at 24.85 °C. NMR solvents were purchased from Deutero GmbH (Kastellaun, Germany) and used as received. ESI-MS spectra were recorded on a Bruker Impact HD Q-TOF spectrometer in the positive ion mode. Thermogravimetric analysis (TGA) was performed using a STA4000 (Perkin Elmer–Waltham, MA, USA) instrument between 25 and 650 °C in a N_2_ atmosphere with a flow rate of 20 mL min^−1^ and a heating rate of 10 °C min^−1^. Powder X-ray diffraction patterns (PXRD) were recorded on a BRUKER D8-Focus Bragg-Brentano X-ray powder diffractometer equipped with a Cu sealed tube (λ = 1.54178Å) at room temperature. The scans were collected in the 2θ range of 5–50°. Experimental and calculated powder patterns from the crystal structures were analysed using Kdif software. A zero point correction was applied to the experimental data for N1 (−0.05), N2 (−0.12) and C1 (0.05). The morphologies of the solid complexes were characterized by scanning electron microscopy (SEM, XL 30 ESEMFEG FEI Company–Hillsboro, ORE, USA). The void volume was visualized and calculated using Digital Discovery Studio 3.5. Brunauer–Emmett–Teller (BET) surface areas were determined by N_2_ adsorption at −196 °C. Prior to the measurements of adsorption/desorption isotherms, the samples were outgassed for 20 h under a pressure of 0.25 mm Hg. Elemental analyses were performed on the apparatus of the German company an Elementar (Langenselbold, Germany), model Vario EL III. 

### 3.2. X-ray Crystallography

X-ray structure determinations for N2, tC1 and [HL3][NO_3_] were performed on a 4-circle Xcalibur EosS2 diffractometer (Agilent Technologies) equipped with a CCD (Charge-coupled Device) detector. X-ray data were collected at room temperature using graphite-monochromated MoKα radiation (*λ* = 0.71073 Å) with the ω-scan technique. The crystal tC1 used for the measurements was identified as a non-merohedral twin with the twin matrix (-1 0 0 0 -1 0 0.997 0.271 1) corresponding to 180° rotation about the (001) reciprocal lattice direction. Olex2 [45] was used as an interface for structure solution, refinement and structural analysis. The crystal structure of tC1 was solved using ShelXT-2015 [46] and was refined with SHELXL-2015 [47] using the diffraction intensity data written in HKLF 5 format.

The X-ray diffraction data for N1 and mC1 were collected on an Oxford Diffraction SuperNova diffractometer equipped with a CuKα radiation source (*λ* = 1.54178 Å) and with a Cryojet cooling system. For data reduction, UB-matrix determination and absorption correction, CrysAlisPro [48] and CrysAlisRed [49] software were applied. Using Olex2 [45], the structures were solved with intrinsic phasing methods employing ShelXT-2015 [46] and were refined by full-matrix least-squares against F^2^ with the help of the SHELXL-2015 [47] refinement package using least squares minimization. All non-hydrogen atoms were refined anisotropically. Some of the hydrogen atoms were derived from a difference Fourier map, and they were refined isotropically. The remaining H-atoms were located in idealized positions by molecular geometry and were refined as riding groups with *U_iso_* (H) = *1.2 U_eq_*(*C*) or *1.5 U_eq_*(*O*). Selected structural parameters are reported in Table 1. The data have been deposited in the Cambridge Crystallographic Data Collection (CCDC), deposition numbers CCDC 1979395-1979398 and 2009855. These data can be obtained free of charge via www.ccdc.cam.ac.uk/data_request/cif, by emailing data_request@ccdc.cam.ac.uk, or by contacting The Cambridge Crystallographic Data Centre, 12, Union Road, Cambridge CB2.

### 3.3. General Preparation of L–L3

The syntheses of 4,4-dimethyl-1-(pyridin-4-yl)-pentane-1,3-dione (L1), 4,4-dimethyl-1-(pyridin-3-yl)-pentane-1,3-dione (L2) and 4,4-dimethyl-1-(pyridin-2-yl)-pentane-1,3-dione (L3) were performed following previously described procedures involving Claisen condensation between appropriate pyridinecarboxylic acid methyl esters and pinacolone in the presence of strong base (NaH) (Figures S1–S3) [24,35].

### 3.4. Synthesis of **[Cu(L1 or 2)_2_]_n_**–N1 and N2

Ligands L1 or L2 (50 mg, 0.24 mmol) in dry THF (5 mL) were added to Na_2_CO_3_ (0.5 g, approximately 5 mmol) suspended in dry THF (10 mL). The mixture was stirred for 1 h before Cu(NO_3_)_2_·5H_2_O (34 mg, 0.12 mmol) (alternatively Cu(CH_3_CO_2_)_2_·H_2_O (24 mg, 0.12 mmol)) in dry THF (5 mL) was added dropwise. The resulting mixture was stirred at 40 °C for 2 h. The excess Na_2_CO_3_ was filtered off, and slow evaporation of the filtrate gave greenish crystals which were collected and washed with EtOH and Et_2_O. Yields were 74% and 71% for N1 and N2, respectively. The coordination polymers N1 and N2 can also be obtained in the form of powders through precipitation with *n*-hexane from the reaction mixture, previously concentrated under reduced pressure. The XRD powder patterns are in full agreement with data obtained from X-ray crystallographic analysis. 

N1: ESI-Q-TOF-HRMS: calcd. for C_24_H_29_N_2_O_4_Cu [Cu(L1)_2_+H]^+^: *m/z* = 472.1418, observed: *m/z* = 472.1421; calcd. for C_36_H_42_N_3_O_6_Cu_2_ [Cu_2_(L1)_3_]^+^: *m/z* = 740.1650, observed: *m/z* = 740.1630; calcd. for C_38_H_48_N_3_O_7_SCu_2_ [Cu_2_(L1)_3_+DMSO]^+^: *m/z* = 818.1789, observed: *m/z* = 818.1767; calcd. for C_60_H_70_N_5_O_10_Cu_3_ [Cu_3_(L1)_5_]^+^: *m/z* = 1211.3000, observed: *m/z* = 1211.2972; Elem. Anal.: calcd. for C_24_H_28_N_2_O_4_Cu: C, 61.07; H, 5.98; N, 5.93. Found: C, 59.81; H, 5.79; N, 5.68 (%).

N2: ESI-Q-TOF-HRMS: calcd. for C_24_H_29_N_2_O_4_Cu [Cu(L2)_2_+H]^+^: *m/z* = 472.1418, observed: *m/z* = 472.1381; calcd. for C_48_H_56_N_4_O_8_Cu_3_ [Cu_3_(L2)_4_]^2+^: *m/z* = 503.5983, observed: *m/z* = 503.5930; calcd. for C_36_H_42_N_3_O_6_Cu_2_ [Cu_2_(L2)_3_]^+^: *m/z* = 738.1660, observed: *m/z* = 738.1687; calcd. for C_72_H_84_N_6_O_12_Cu_4_ [Cu_4_(L2)_6_]^2+^: *m/z* = 739.1658, observed: *m/z* = 739.1658; calcd. for C_48_H_57_N_4_O_8_Cu_2_ [Cu_2_(L2)_4_+H]^+^: *m/z* = 945.2757, observed: *m/z* = 945.2758; calcd. for C_96_H_112_N_8_O_16_Cu_5_ [Cu_5_(L2)_8_]^2+^: *m/z* = 975.7333, observed: *m/z* = 975.7285; calcd. for C_60_H_70_N_5_O_10_Cu_3_ [Cu_3_(L2)_5_]^+^: *m/z* = 1211.3000, observed: *m/z* = 1211.2975; Elem. Anal.: calcd. for C_24_H_28_N_2_O_4_Cu: C, 61.07; H, 5.98; N, 5.93. Found: C, 60.95; H, 5.84; N, 5.82 (%).

### 3.5. Synthesis of Cu(L3)_2_–C1

A solution of L3 (50 mg, 0.24 mmol) and Cu(CH_3_COO)_2_·H_2_O (24 mg, 0.12 mmol) in THF/MeOH (1:1, *v/v,* 10 mL) was stirred at room temperature for 24 h, after which DMSO (1 mL) was added. The mixture was left in air for 7 days at room temperature. Dark green crystals tC1 of the triclinic form slowly deposited. The product was filtered off and washed with EtOH and Et_2_O. Single crystals of mC1 as a monoclinic form were obtained by diffusion of *tert*-butyl methyl ether vapours into a THF solution of the complex. Yield was 85%. ESI-Q-TOF-HRMS: calcd. for C_24_H_28_N_2_O_4_CuNa [Cu(L3)_2_+Na]^+^: *m/z* = 494.1237, observed: *m/z* = 494.1242; calcd. for C_48_H_56_N_4_O_8_Cu_2_Na {[Cu(L3)_2_]_2_+Na}^+^: *m/z* = 967.2577, observed: *m/z* = 967.2549; Elem. Anal.: calcd. for C_24_H_28_N_2_O_4_Cu: C, 61.07; H, 5.98; N, 5.93. Found: C, 60.86; H, 5.79; N, 5.78 (%).

## 4. Conclusions

In summary, we have successfully generated and fully analysed three structurally distinct metallosupramolecular frameworks employing Cu(II) ions and ambidentate pyridyl-β-diketonate ligands. The structural features, stability and morphology of these coordination compounds were determined by single-crystal and powder XRD, TGA, TOF-MS, SEM and elemental analysis. This work demonstrates that variations of the *N*-atom position in the pyridyl ring are critical to the type of metal-organic assembly generated. In addition to significant differences in topology and dimensionality, disparities in physical properties between studied metal-organic assemblies were observed, confirming the determining role of the *N*-atom position not only on the structure but also on the potential function of these materials.

## Figures and Tables

**Table 1:** Crystal data and structure refinement for coordination compounds N1–N2 and C1 and for ligand [HL3][NO_3_].

Parameters	N1–[Cu(L1)_2_]_n_	N2–[Cu(L2)_2_]_n_	tC1–Cu(L3)_2_	mC1–Cu(L3)_2_	[HL3][NO_3_]
CCDC code	1979395	1979396	2009855	1979398	1979397
Empirical formula	C_24_H_28_CuN_2_O_4_	C_24_H_28_CuN_2_O_4_	C_24_H_28_CuN_2_O_4_	C_24_H_28_CuN_2_O_4_	C_12_H_16_N_2_O_5_
Formula weight	472.02	472.02	472.02	472.02	268.27
Temperature/K	130.6(3)	293(2)	293(2)	133(2)	293(2)
Crystal system	monoclinic	monoclinic	triclinic	Monoclinic	Monoclinic
Space group	P2_1_/n	P2_1_/n	P-1	P2_1_/n	P2_1_/c
a/Å	9.67360(10)	11.5058(3)	6.3111(8)	13.1273(6)	11.3313(4)
b/Å	12.46690(10)	9.3195(2)	9.4952(11)	6.0457(2)	10.0225(4)
c/Å	9.73330(10)	12.3363(3)	10.5041(13)	14.2591(5)	11.9033(5)
α/°	90	90	94.356(10)	90	90
*β*/°	90.0270(10)	108.256(3)	106.293(11)	92.322(3)	91.277(3)
γ/°	90	90	98.971(10)	90	90
Volume/Å^3^	1173.83(2)	1256.22(6)	592.01(13)	1130.73(8)	1351.50(9)
Z	2	2	1	2	4
ρ_calc_g/cm^3^	1.335	1.248	1.324	1.386	1.318
μ/mm^-1^	1.569	0.898	0.953	1.629	0.103
F(000)	494.0	494.0	247.0	494.0	568.0
Crystal size/mm^3^	0.62 × 0.36 × 0.34	0.15 × 0.1 × 0.05	0.3 × 0.05 × 0.03	0.2 × 0.1 × 0.02	0.2 × 0.15 × 0.1
Radiation	Cu Kα (λ = 1.54184)	MoKα (λ = 0.71073)	MoKα (λ = 0.71073)	CuKα (λ = 1.54184)	MoKα (λ = 0.71073)
2Θ range for data collection/°	11.578 to 152.524	8.218 to 53.446	8.528 to 50.042	8.978 to 152.556	6.46 to 59.074
Index ranges	−10 ≤ h ≤ 12, −15 ≤ k ≤ 15, −12 ≤ l ≤ 10	−14 ≤ h ≤ 14, −11 ≤ k ≤ 11, −15 ≤ l ≤ 15	−7 ≤ h ≤ 7, −11 ≤ k ≤ 11, −6 ≤ l ≤ 12	−15 ≤ h ≤ 16, −7 ≤ k ≤ 7, −17 ≤ l ≤ 12	−15 ≤ h ≤ 15, −13 ≤ k ≤ 13, −14 ≤ l ≤ 14
Reflections collected	21,676	56,535	2094	4407	50,829
Independent reflections	2428 (R_int_ = 0.0320, R_sigma_ = 0.0123)	2662 (R_int_ = 0.0444, R_sigma_ = 0.0160)	2094 (R_int_ = merged, R_sigma_ = 0.0956)	2299 (R_int_ = 0.0209, R_sigma_ = 0.0254)	3209 (R_int_ = 0.0762, R_sigma_ = 0.0317)
Data/restraints/parameters	2428/0/188	2662/0/166	2094/0/146	2299/0/176	3209/0/214
Goodness-of-fit on F^2^	1.083	1.038	1.096	1.074	1.098
Final R indexes [I ≥ 2σ (I)]	R_1_ = 0.0321, wR_2_ = 0.0860	R_1_ = 0.0359, wR_2_ = 0.0928	R_1_ = 0.0701, wR_2_ = 0.1488	R_1_ = 0.0460, wR_2_ = 0.1288	R_1_ = 0.0752, wR_2_ = 0.1715
Final R indexes (all data)	R_1_ = 0.0325, wR_2_ = 0.0862	R_1_ = 0.0448, wR_2_ = 0.0976	R_1_ = 0.1134, wR_2_ = 0.1698	R_1_ = 0.0517, wR_2_ = 0.1357	R_1_ = 0.1101, wR_2_ = 0.1906
Largest diff. peak/hole/e Å^-3^	0.32/-0.38	0.37/-0.29	0.37/-0.49	0.92/-0.55	0.61/-0.17

## Supplementary Materials

Supplementary materials can be found at https://www.mdpi.com/1422-0067/21/17/6171/s1. Scheme S1: General scheme for the preparation of ligands L1–L3. Figure S1: 1H NMR spectrum (300 MHz, CDCl3) of L1, Figure S2: 1H NMR spectrum (300 MHz, CDCl3) of L2, Figure S3: 1H NMR spectrum (300 MHz, CDCl3) of L3, Figure S4: ESI-Q-TOF-HRMS spectrum of the polymer N1, Figure S5: The thermogravimetric analysis (TGA) curve for N1, Figure S6: Scanning electron microscopy (SEM) images of crystalline fibre of the polymer N1, Figure S7: ESI-Q-TOF-HRMS spectrum of the polymer N2, Figure S8: The thermogravimetric analysis (TGA) curve for N2, Figure S9: Scanning electron microscopy (SEM) images of crystalline fibre of the polymer N2; Figure S10: ESI-Q-TOF-HRMS spectrum of the complex C1; Figure S11: The thermogravimetric analysis (TGA) curve for C1, Figure S12: Scanning electron microscopy (SEM) images of crystals C1, Figure S13: Calculated void volume of the pore in the structure of polymer N1, Table S1: All parameters related to the measurement of N2 sorption, Figure S14: N2 adsorption–desorption isotherms of coordination polymers N1–N2 and complex C1, Table S2: N2 adsorption–desorption data of coordination polymers N1–N2 and complex C1.

## Abbreviations

calcd.	calculated
CCD	Charge-coupled Device
XRD	X-Ray diffraction
PXRD	powder X-Ray diffraction
TGA	thermogravimetric analysis
TOF-MS	time-of-flight mass spectrometry
SEM	scanning electron microscopy
LFD	large field detector
THF	tetrahydrofuran
MeOH	methanol
NaH	sodium hydride
BET	Brunauer–Emmett–Teller isotherm
CPs	coordination polymers
