# Supplementary material for: Intrinsic Effect of Pyridine-N-Position on Structural Properties of Cu-Based Low-Dimensional Coordination Frameworks
Source: Int J Mol Sci. 2020 Aug 26;21(17):6171. doi: 10.3390/ijms21176171 (PMC7503679; doi:10.3390/ijms21176171)
Supplement: Supplementary file 1 [file ijms-21-06171-s001.pdf]

Supporting Information for

Intrinsic effect of pyridine-N-position on structural properties of Cu-based low-dimensional coordination frameworks

*Anna Walczak, Gracjan Kurpik, and Artur R. Stefankiewicz**

Table of contents

1. Synthesis of ligands 2
    1.1. Synthesis of ligand L1 2
    1.2. Synthesis of ligand L2 3
    1.3. Synthesis of ligand L3 4
2. Experimental data for Cu(II) compounds 5
    2.1. Polymer $[Cu(L1)_2]_n$ (N1) 5
        2.1.1. Mass spectrometry 5
        2.1.2.TGA analysis 6
        2.1.3. SEM images 7
    2.2. Polymer $[Cu(L2)_2]_n$ (N2) 7
        2.2.1. Mass spectrometry 7
        2.2.2. TGA analysis 8
        2.2.3. SEM images 9
2.3. Complex $Cu(L3)_2$ (C1) 9
        2.3.1. Mass spectrometry 9
        2.3.2. TGA analysis 10
        2.3.3. SEM images 11
3. Void volume of the pore in N1 12
4. Gas sorption studies .... **Error! Bookmark not defined.**
5. References 14

1. Synthesis of ligands

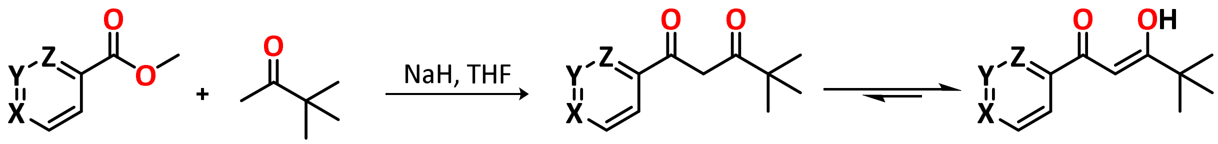

L1 X = N, Y = Z = CH; **L2** Y = N, X = Z = CH; **L3** Z = N; X = Y = CH

Scheme S1. General scheme for the preparation of ligands **L1** – **L3**.

The ligands **L1** – **L3** were prepared by Claisen condensation, following a literature procedure and involving the reaction between the appropriate methyl esters and 3,3-dimethyl-2-butanone in the presence of sodium hydride.[1-2]

1.1. Synthesis of ligand L1

The reaction of methyl isonicotinate (5.0 g, 36.5 mmol) with 3,3-dimethyl-2-butanone (6.4 mL, 51.1 mmol) gave **L1** in the form of a brown oil. Yield: 5.8 g, 78%.

^{1}H NMR (300 MHz, $CDCl_3$) δ = 16.09 (s, 1H, H^4), 8.75 (dd, *J* = 5.8, 1.6 Hz, 2H, H^2), 7.69 (dd, *J* = 6.1, 1.8 Hz, 2H, H^1), 6.34 (s, 1H, H^3), 4.19 (s, $H^{3'}$), 1.26 (s, 9H, H^5).

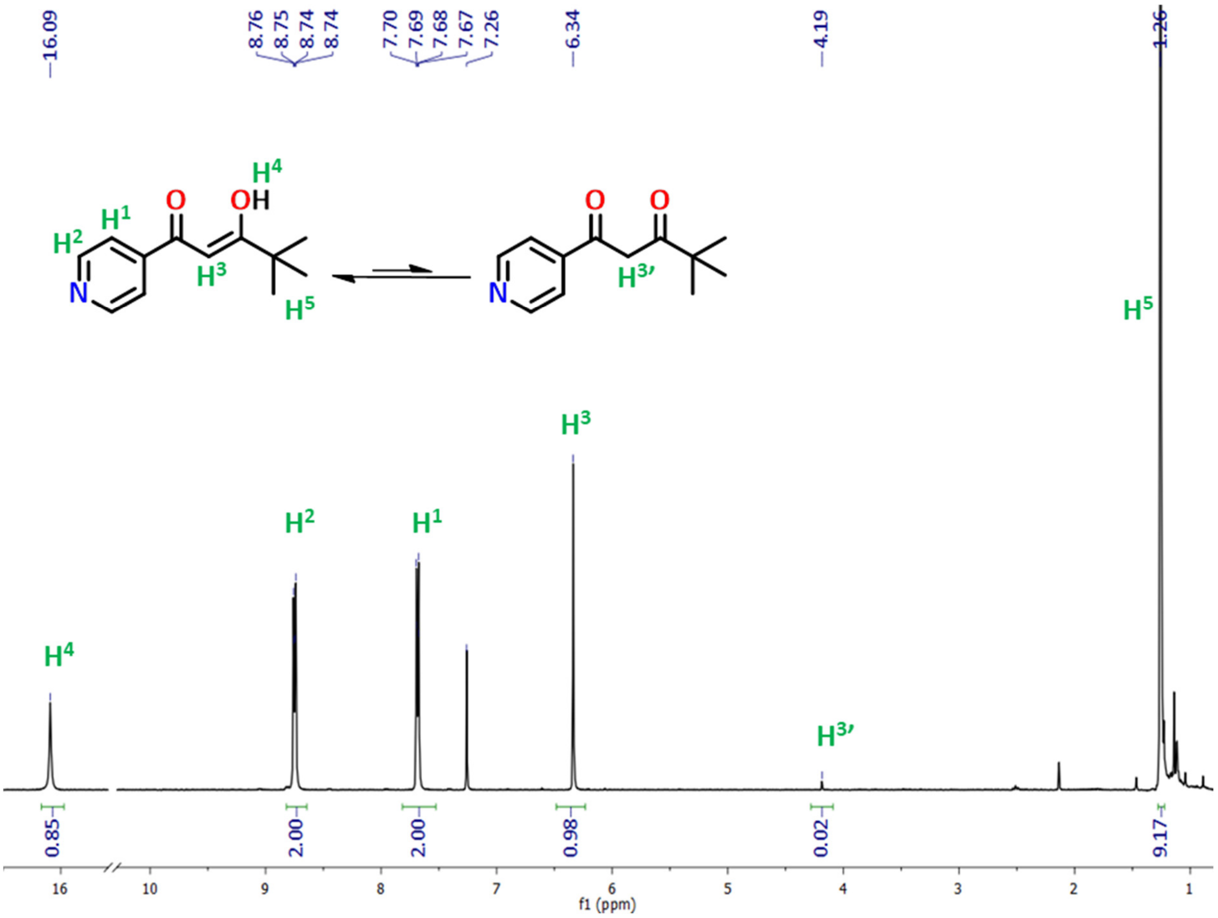

Figure S1. ^{1}H NMR spectrum (300 MHz, $CDCl_3$) of **L1**.

1.2. Synthesis of ligand L2

The reaction of methyl nicotinate (5.0 g, 36.5 mmol) with 3,3-dimethyl-2-butanone (6.4 mL, 51.1 mmol) gave **L2** in the form of a orange oil. Yield: 6.2 g, 83%.

^{1}H NMR (300 MHz, $CDCl_3$) δ = 16.32 (s, 1H, H^6), 9.08 (dd, *J* = 1.8, 0.9 Hz, 1H, H^1), 8.73 (dd, *J* = 4.8, 1.6 Hz, 1H, H^2), 8.17 (dt, *J* = 8.0, 2.0 Hz, 1H, H^4), 7.40 (ddt, *J* = 7.9, 5.0, 1.1 Hz, 1H, H^3), 6.31 (s, 1H, H^5), 4.21 (s, $H^{5'}$), 1.26 (s, 9H, H^7).

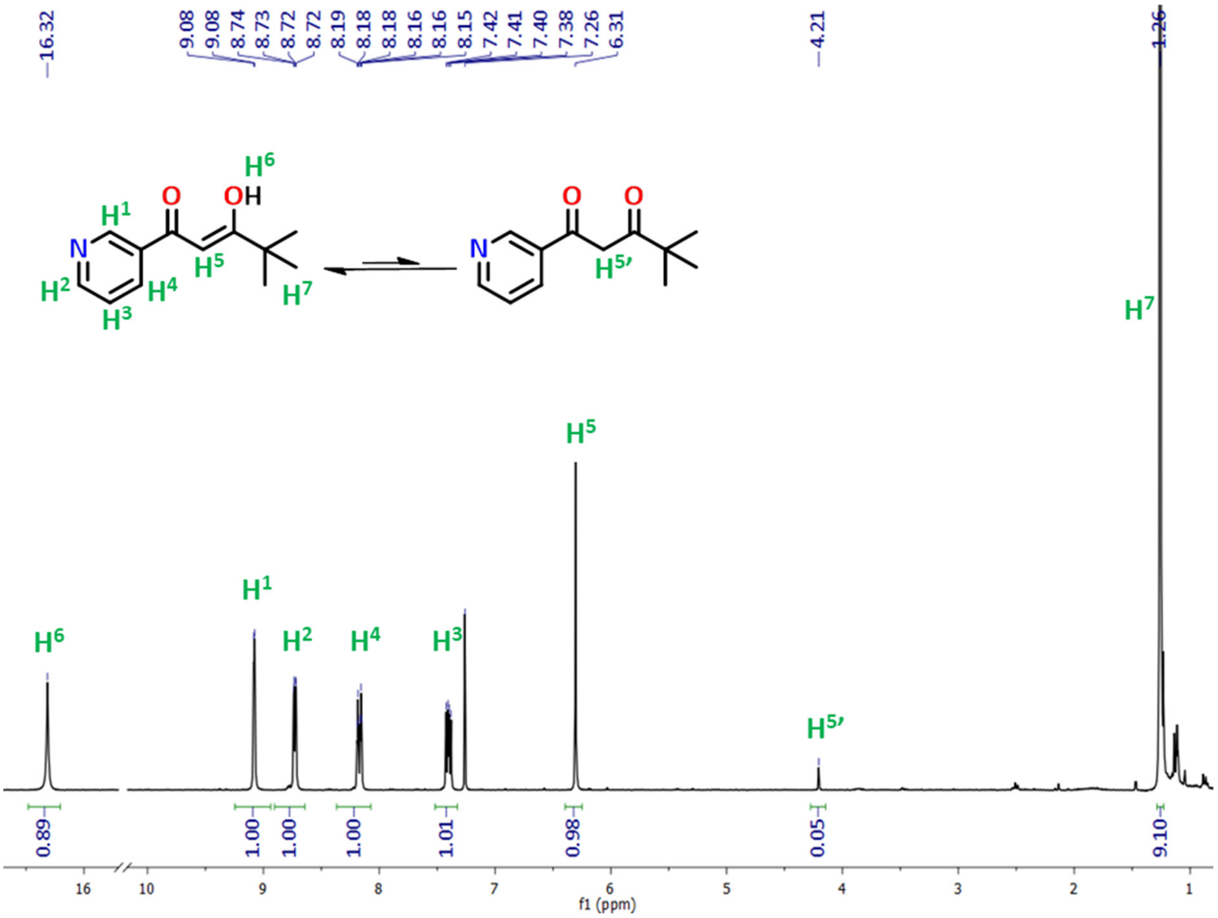

Figure S2. ^{1}H NMR spectrum (300 MHz, $CDCl_3$) of **L2**.

1.3. Synthesis of ligand L3

The reaction of methyl picolinate (5.0 g, 36.5 mmol) with 3,3-dimethyl-2-butanone (6.4 mL, 51.1 mmol) gave **L3** in the form of a brown oil. Yield: 6.4 g, 86%.

^{1}H NMR (300 MHz, $CDCl_3$) δ = 16.12 (s, 1H, H^6), 8.65 (ddd, J = 4.7, 1.8, 0.9 Hz, 1H, H^1), 8.06 (dt, J = 7.9, 1.1 Hz, 1H, H^4), 7.81 (td, J = 7.7, 1.7 Hz, 1H, H^2), 7.38 (ddd, J = 7.6, 4.7, 1.2 Hz, 1H, H^3), 6.97 (s, 1H, H^5), 4.36 (s, $H^{5'}$), 1.26 (s, 9H, H^7).

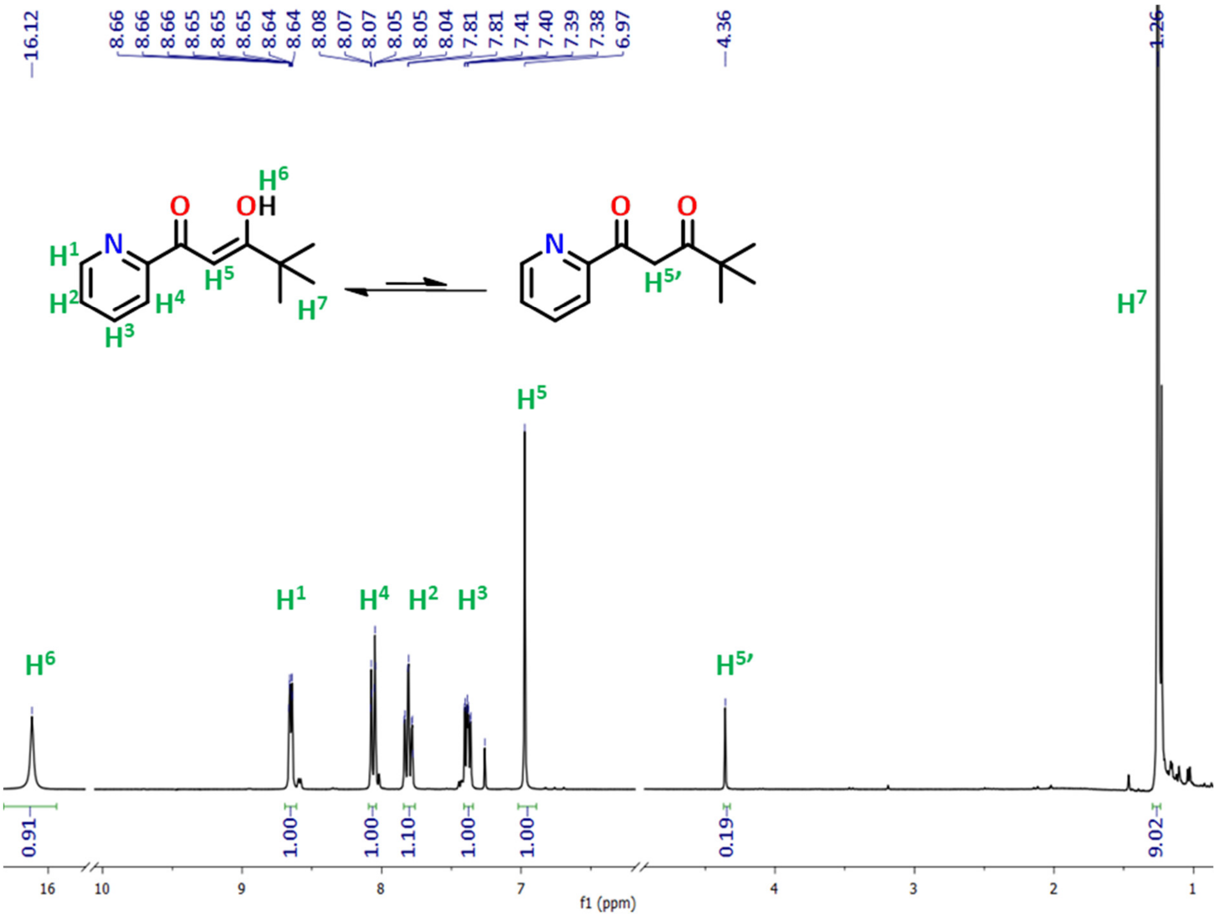

Figure S3. ^{1}H NMR spectrum (300 MHz, $CDCl_3$) of **L3**.

2. Experimental data for Cu(II) compounds

2.1. Polymer $[Cu(L1)_2]_n$ (N1)

2.1.1. Mass spectrometry

ESI-Q-TOF-HRMS calcd. for $C_{24}H_{29}N_2O_4Cu$ $[Cu(\mathbf{L1})_2+H]^+$: *m*/*z* = 472.1418, observed: *m*/*z* = 472.1421; calcd. for $C_{36}H_{42}N_3O_6Cu_2$ $[Cu_2(\mathbf{L1})_3]^+$: *m*/*z* = 740.1650, observed: *m*/*z* = 740.1630; calcd. for $C_{38}H_{48}N_3O_7SCu_2$ $[Cu_2(\mathbf{L1})_3+DMSO]^+$: *m*/*z* = 818.1789, observed: *m*/*z* = 818.1767; calcd. for $C_{60}H_{70}N_5O_{10}Cu_3$ $[Cu_3(\mathbf{L1})_5]^+$: *m*/*z* = 1211.3000, observed: *m*/*z* = 1211.2972.

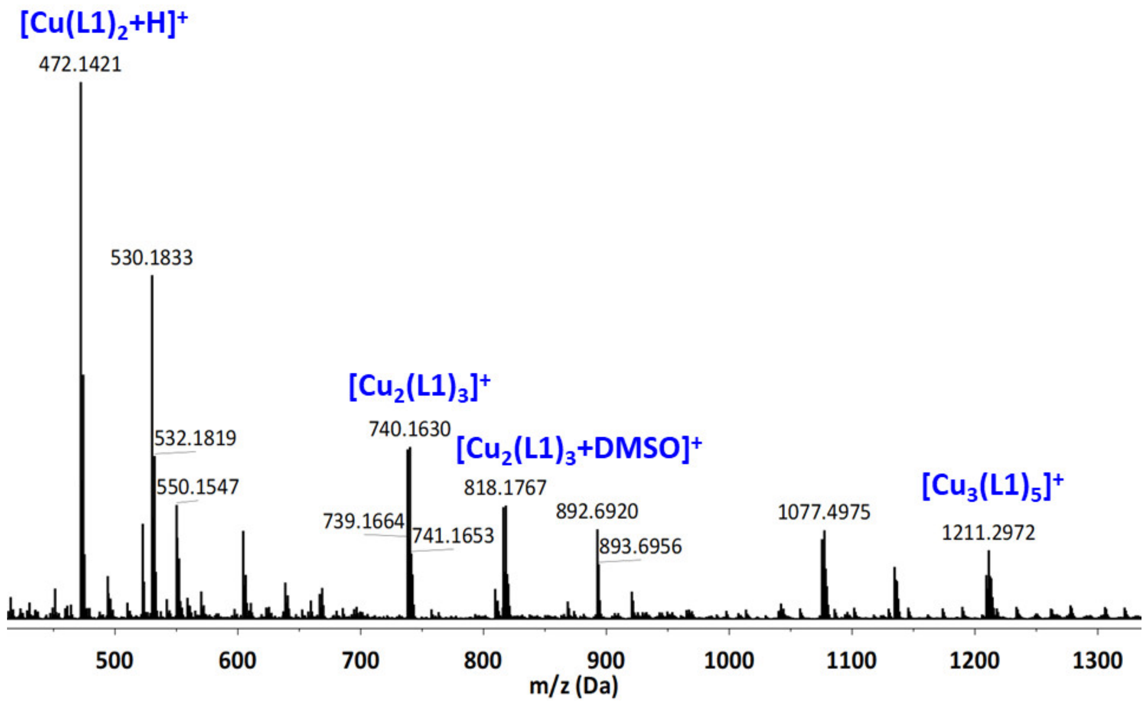

Figure S4. ESI-Q-TOF-HRMS spectrum of the polymer **N1**.

2.1.2. TGA analysis

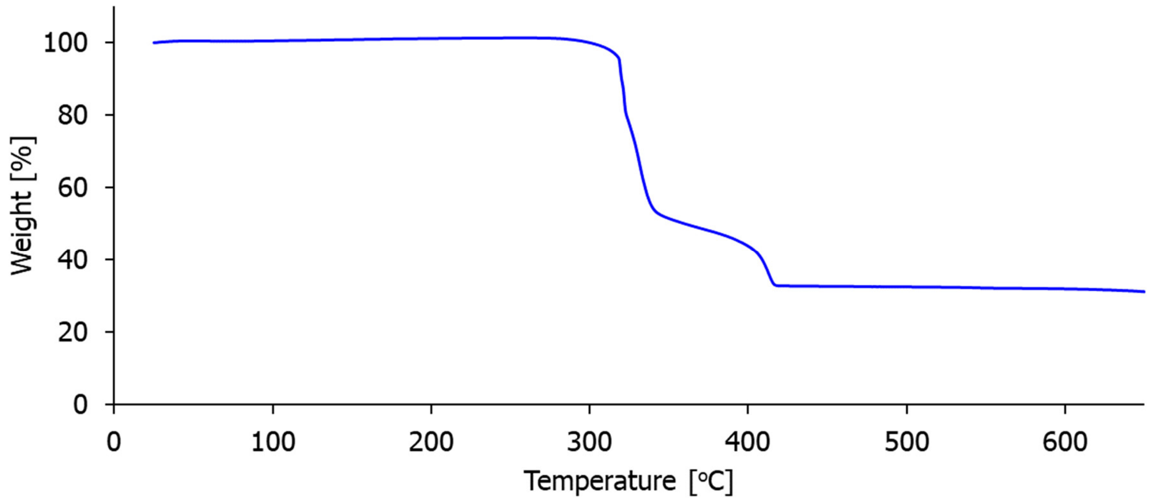

Figure S5. The thermogravimetric analysis (TGA) curve for **N1**.

2.1.3. SEM images

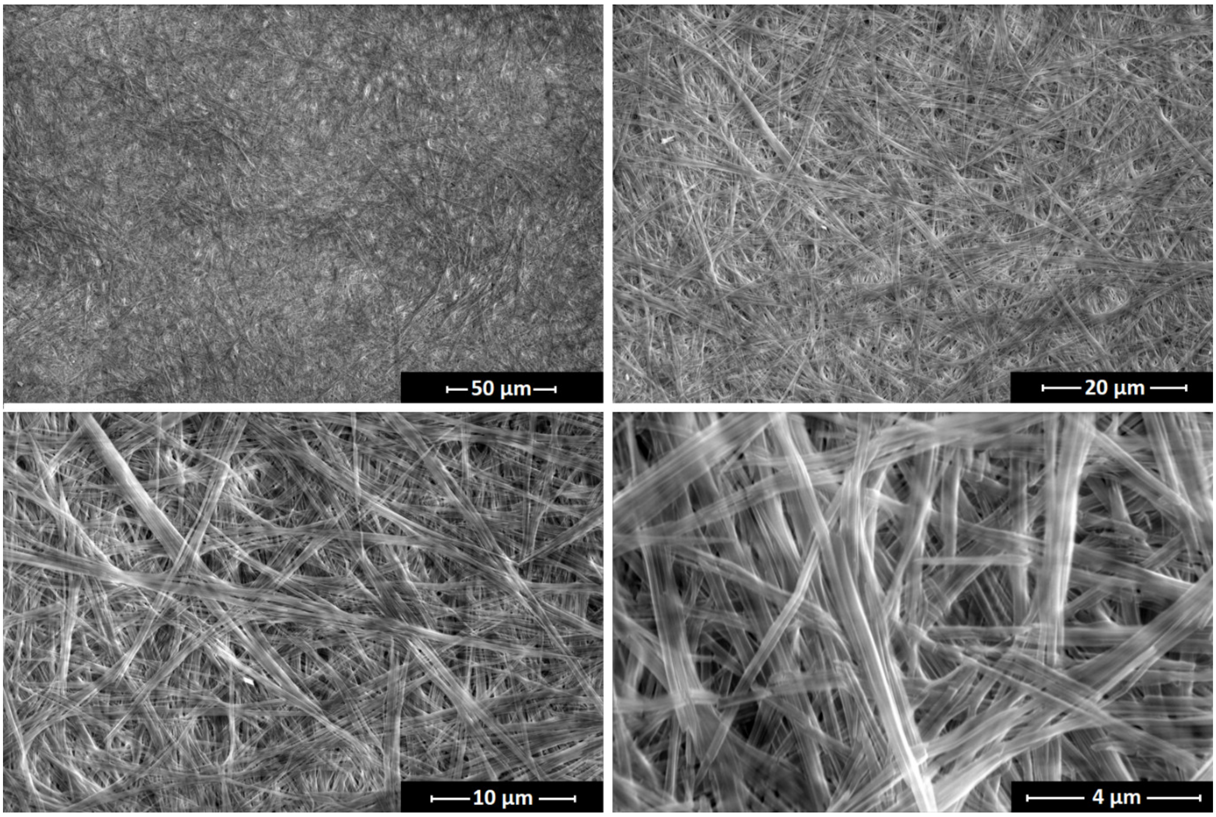

Figure S6. Scanning electron microscopy (SEM) images of crystalline fiber of the polymer **N1**.

2.2. Polymer [Cu(L2)$_2$]$_n$ (N2)

2.2.1. Mass spectrometry

ESI-Q-TOF-HRMS calcd. for $C_{24}H_{29}N_2O_4Cu$ [Cu(**L2**)$_2$+H]$^+$: *m*/*z* = 472.1418, observed: *m*/*z* = 472.1381; calcd. for $C_{48}H_{56}N_4O_8Cu_3$ [Cu$_3$(**L2**)$_4$]$^{2+}$: *m*/*z* = 503.5983, observed: *m*/*z* = 503.5930; calcd. for $C_{36}H_{42}N_3O_6Cu_2$ [Cu$_2$(**L2**)$_3$]$^+$: *m*/*z* = 738.1660, observed: *m*/*z* = 738.1687; calcd. for $C_{72}H_{84}N_6O_{12}Cu_4$ [Cu$_4$(**L2**)$_6$]$^{2+}$: *m*/*z* = 739.1658, observed: *m*/*z* = 739.1658; calcd. for $C_{48}H_{57}N_4O_8Cu_2$ [Cu$_2$(**L2**)$_4$+H]$^+$: *m*/*z* = 945.2757, observed: *m*/*z* = 945.2758; calcd. for $C_{96}H_{112}N_8O_{16}Cu_5$ [Cu$_5$(**L2**)$_8$]$^{2+}$: *m*/*z* = 975.7333, observed: *m*/*z* = 975.7285; calcd. for $C_{60}H_{70}N_5O_{10}Cu_3$ [Cu$_3$(**L2**)$_5$]$^+$: *m*/*z* = 1211.3000, observed: *m*/*z* = 1211.2975.

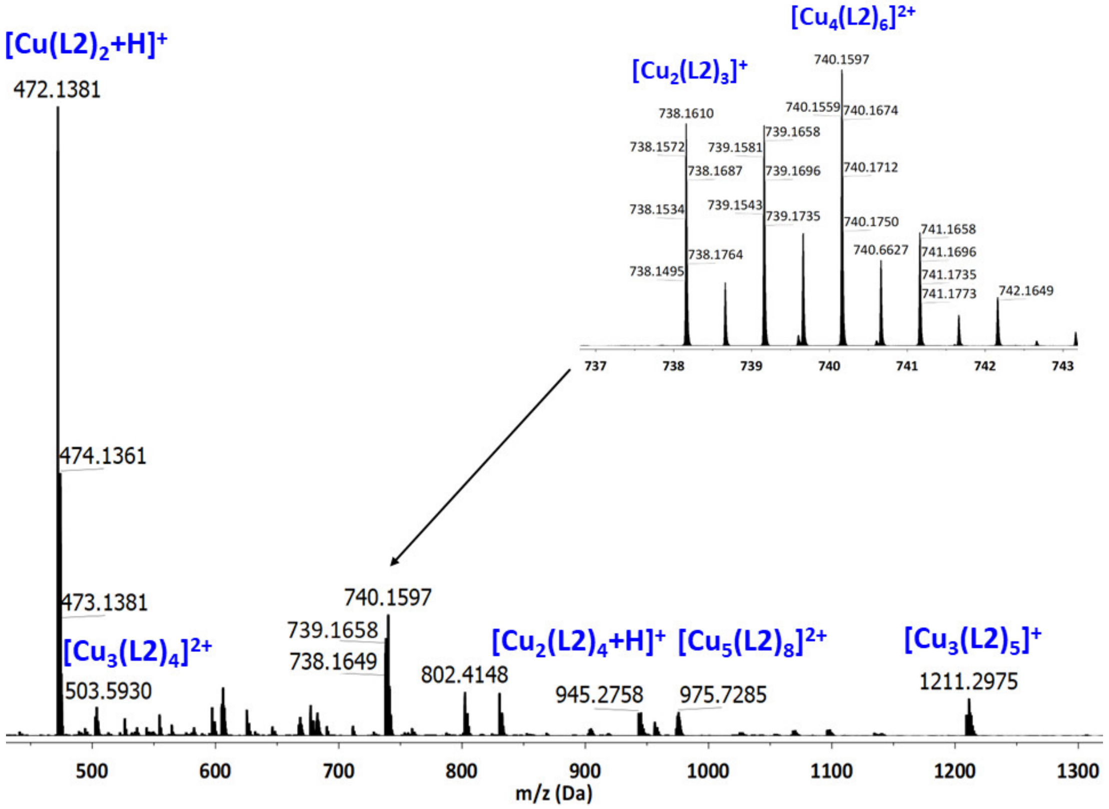

Figure S7. ESI-Q-TOF-HRMS spectrum of the polymer **N2**.

2.2.2. TGA analysis

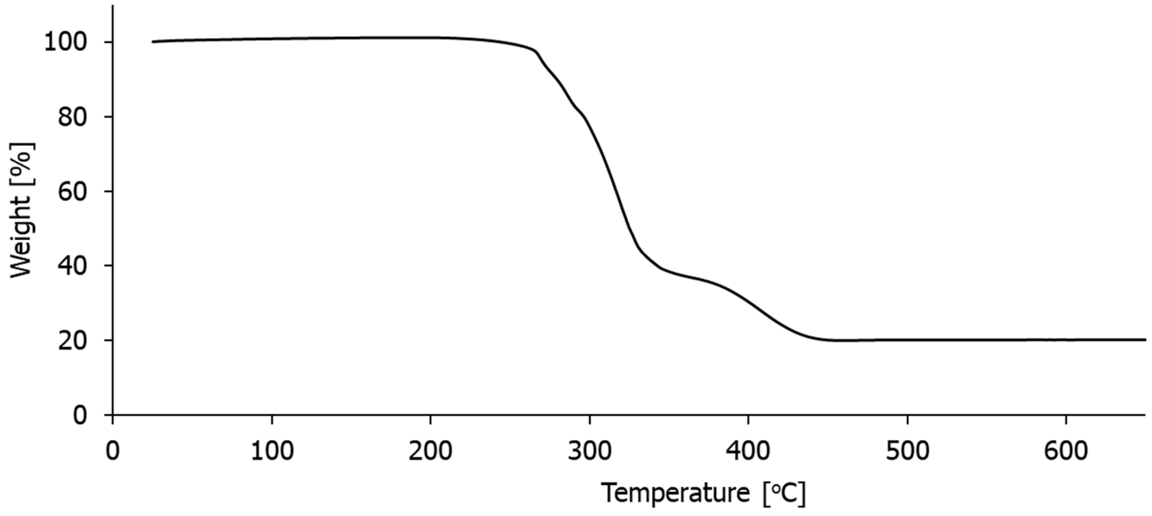

Figure S8. The thermogravimetric analysis (TGA) curve for **N2**.

2.2.3. SEM images

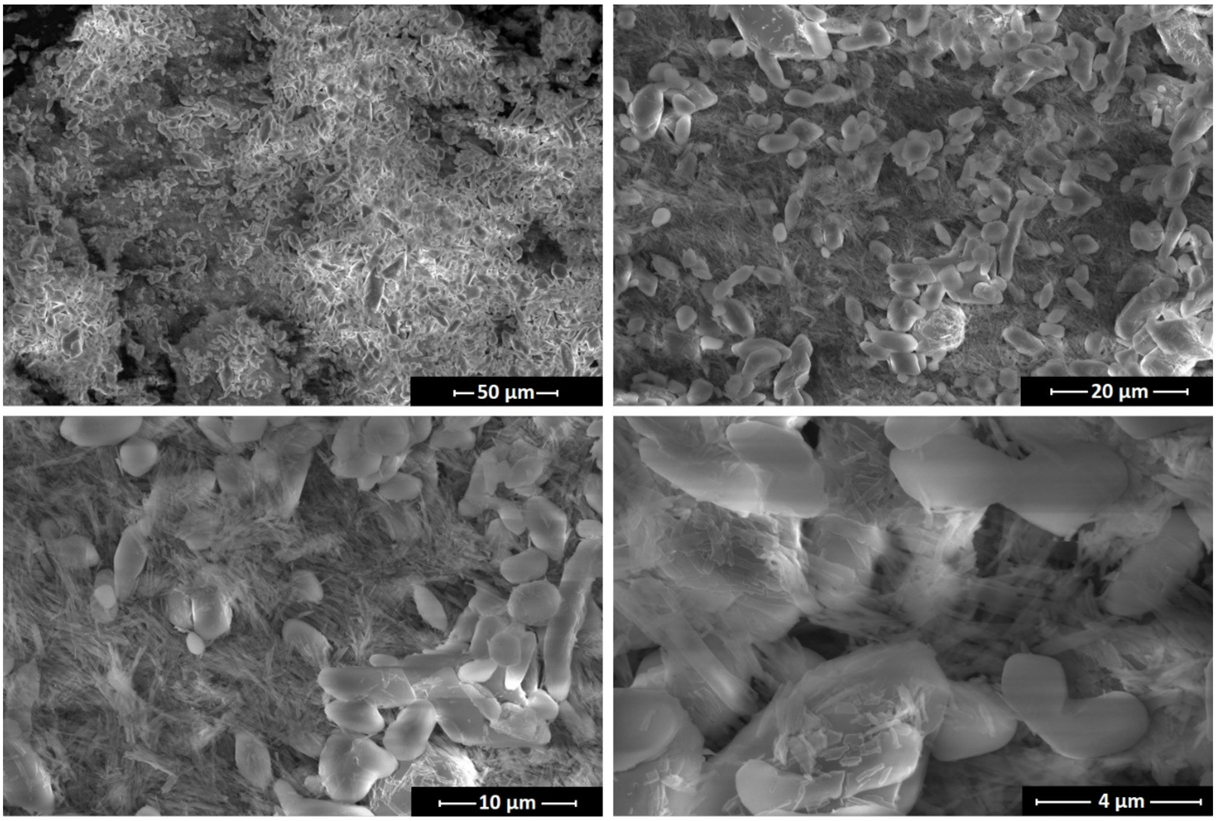

Figure S9. Scanning electron microscopy (SEM) images of crystalline fiber of the polymer **N2**.

2.3. Complex Cu(L3)$_2$ (C1)

2.3.1. Mass spectrometry

ESI-Q-TOF-HRMS calcd. for $C_{24}H_{28}N_2O_4CuNa$ [Cu(**L3**)$_2$+Na]$^+$: *m*/*z* = 494.1237, observed: *m*/*z* = 494.1242; calcd. for $C_{48}H_{56}N_4O_8Cu_2Na$ {[Cu(**L3**)$_2$]$_2$+Na}$^+$: *m*/*z* = 967.2577, observed: *m*/*z* = 967.2549.

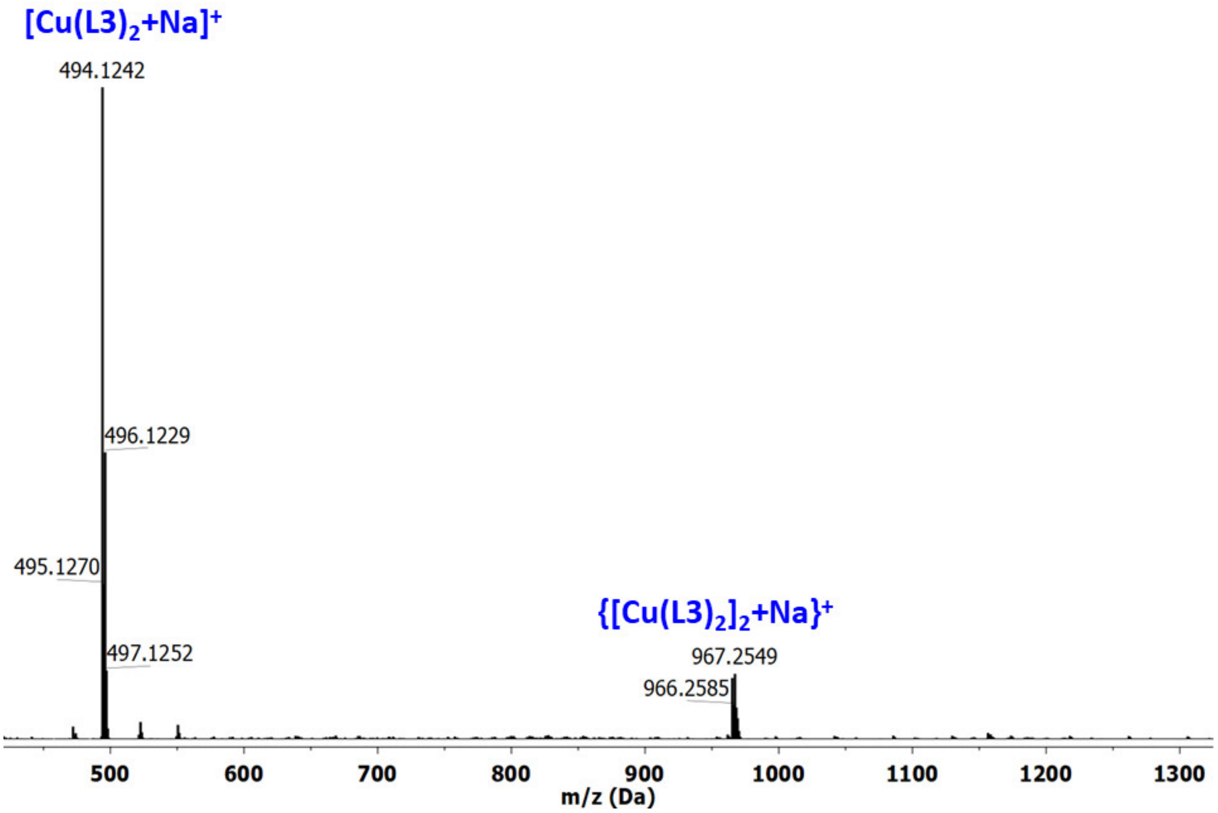

Figure S10. ESI-Q-TOF-HRMS spectrum of the complex **C1**.

2.3.2. TGA analysis

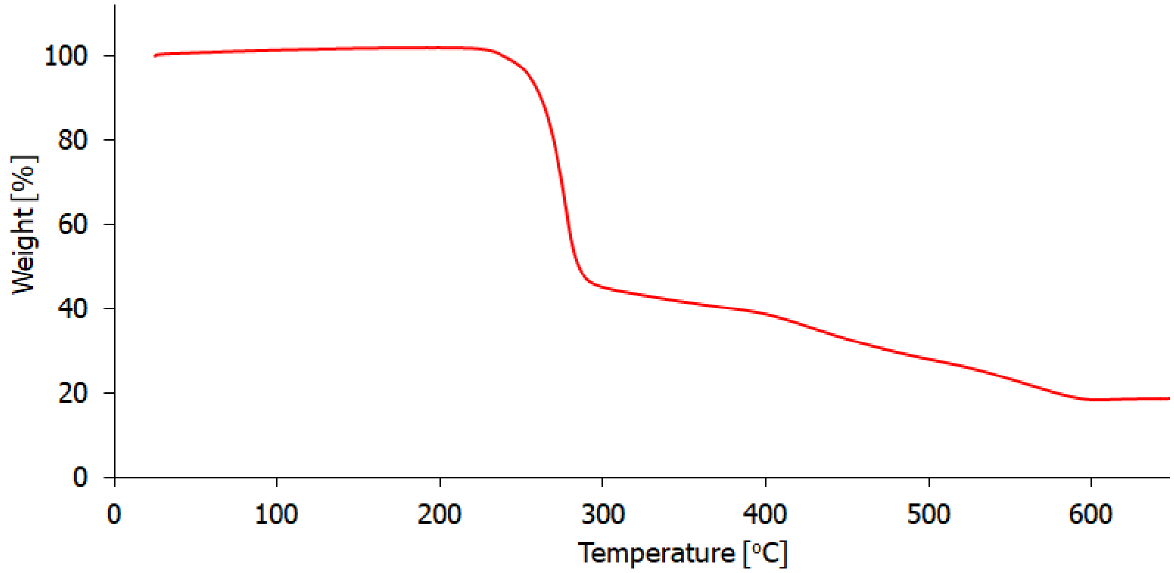

Figure S11. The thermogravimetric analysis (TGA) curve for C1.

2.3.3. SEM images

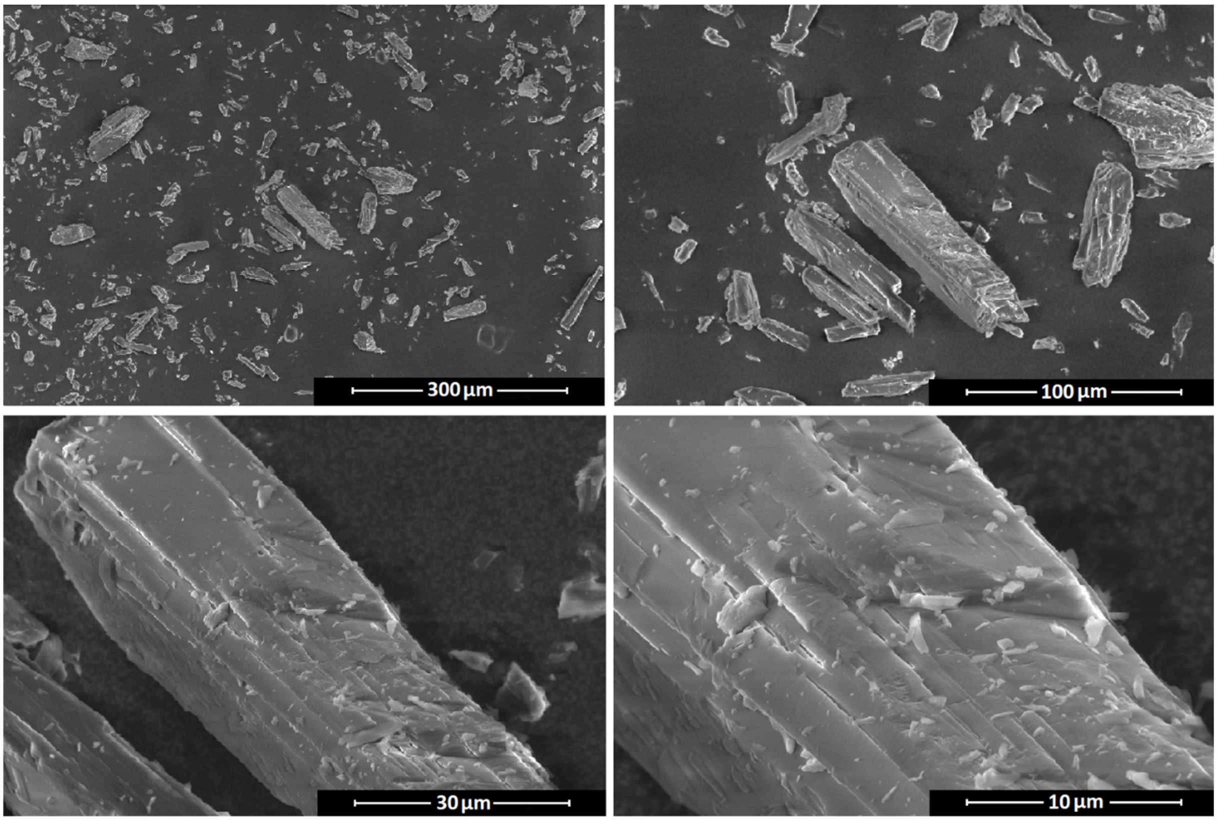

Figure S12. Scanning electron microscopy (SEM) images of crystals **C1**.

3. Void volume of the pore in N1

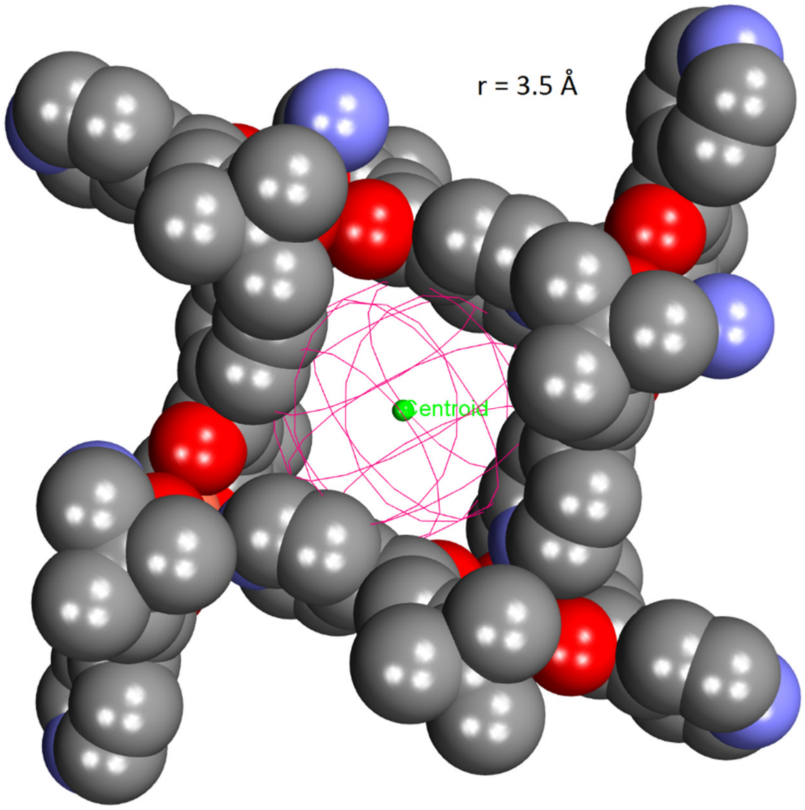

Figure S13. Calculated void volume of the pore in the structure of polymer **N1**.

4. Gas sorption studies

Table S1. All parameters related to the measurement of N_2 sorption.

	**N1**	**N2**	**C1**
**BET surface area [m²/g]**	4.3182	1.9048	2.1630
**average pore volume [cm³/g]**	0.0063	0.0032	0.0059
**average pore diameter [nm]**	5.8408	6.7254	10.8153

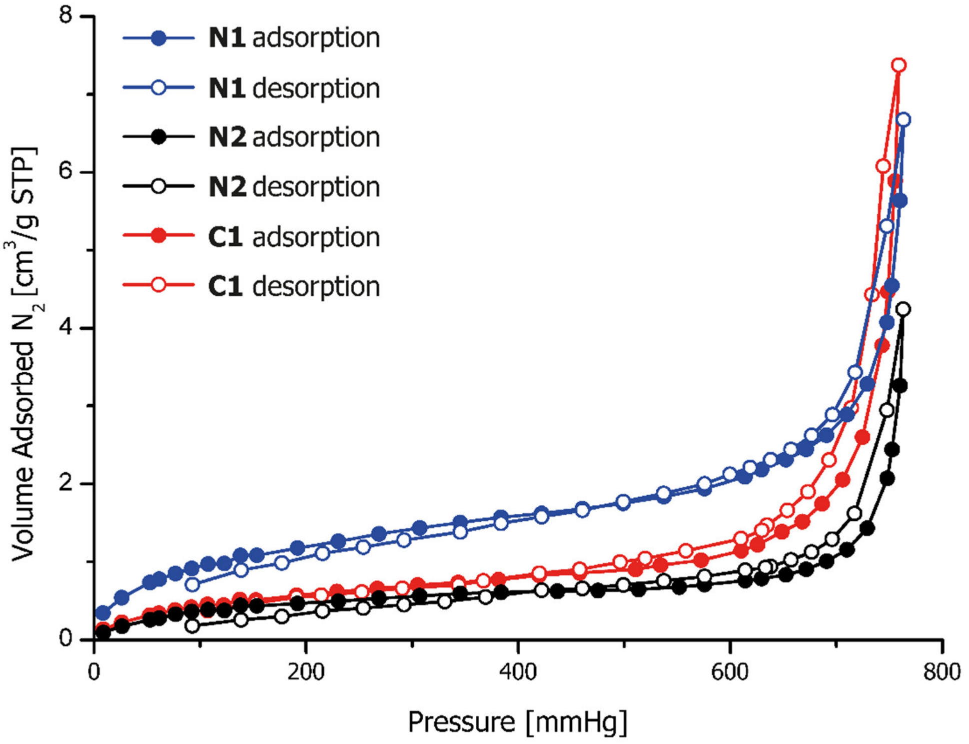

Figure S14. N_2 adsorption–desorption isotherms of coordination polymers **N1-N2** and complex **C1**.

Table S2. N_2 adsorption–desorption data of coordination polymers **N1-N2** and complex **C1**.

N1		N2		C1	
pressure [mmHg]	volume adsorbed N_2 [cm^3/g STP]	pressure [mmHg]	volume adsorbed N_2 [cm^3/g STP]	pressure [mmHg]	volume adsorbed N_2 [cm^3/g STP]
8,3778	0,3436	8,7916	0,0944	8,5847	0,1349
26,2247	0,5414	26,4264	0,1750	26,1937	0,2243
52,6976	0,7349	52,9045	0,2572	52,4908	0,3152
61,4271	0,7768	61,4374	0,2784	61,0755	0,3418
76,7968	0,8493	76,7968	0,3285	76,3831	0,3842
92,1561	0,9159	92,1768	0,3623	91,6287	0,4191
107,5103	0,9706	107,5672	0,3873	106,8950	0,4564
122,9266	0,9798	122,9472	0,3768	122,2543	0,4462
138,1515	1,0805	138,1825	0,4433	137,3603	0,5142
153,5936	1,0840	153,6142	0,4344	152,7041	0,5108
192,0075	1,1766	192,0178	0,4677	190,8801	0,5701
230,3800	1,2609	230,3386	0,4962	229,0458	0,6177
268,6594	1,3575	268,7112	0,5333	267,1546	0,6569
307,0889	1,4338	307,0837	0,5653	305,2789	0,7029
345,4045	1,5026	345,4563	0,5958	343,4912	0,7355
383,7823	1,5705	383,8546	0,6082	381,5482	0,7697

422,1960	1,6190	436,9142	0,6227	419,6828	0,8259
460,4704	1,6801	475,2557	0,6317	457,9985	0,8595
498,8946	1,7496	513,5817	0,6461	510,6340	0,9025
537,2672	1,8349	551,9025	0,6777	534,1127	0,9587
575,6397	1,9387	575,6914	0,7059	572,4179	1,0209
613,9605	2,0915	614,0639	0,7575	610,2888	1,1414
629,4233	2,1810	629,4130	0,7848	625,8499	1,2197
652,4882	2,3085	652,4365	0,8343	648,6252	1,3861
671,5194	2,4428	671,5710	0,9035	667,8890	1,5157
690,7470	2,6213	690,8090	1,0092	686,5219	1,7441
709,8920	2,8867	709,9952	1,1564	705,9616	2,0524
729,0781	3,2804	729,0264	1,4305	724,5273	3,7810
747,9023	4,0765	747,9386	2,0705	743,1446	3,7810
752,3448	4,5484	752,4533	2,4389	748,4506	4,4692
760,0038	5,6374	760,1122	3,2595	755,3495	5,8894
763,2617	6,6777	762,9824	4,2439	758,9179	7,3765
747,6439	5,3114	747,8093	2,9441	744,1892	6,0811
717,7525	3,4314	717,1215	1,6192	733,6290	4,4322
696,3631	2,8848	696,1047	1,2907	714,0654	2,9733
676,6909	2,6195	676,5873	1,1256	693,0432	2,3032
657,3495	2,4392	657,2770	1,0257	673,2726	1,8994
638,0597	2,3080	638,0597	0,9404	653,7812	1,6610
618,8011	2,2083	633,2657	0,9279	634,5742	1,4707
599,5890	2,1220	614,1157	0,8914	629,7338	1,4013
575,6914	2,0000	575,7535	0,8117	610,1130	1,2980
537,1638	1,8780	537,2672	0,7570	558,0877	1,1436
498,9981	1,7734	498,9981	0,7016	519,7875	1,0453
460,5686	1,6609	460,5377	0,6601	496,0504	0,9962
422,2012	1,5774	422,2012	0,6371	457,9881	0,9034
383,8753	1,4927	369,1934	0,5502	419,9258	0,8556
345,5648	1,3829	330,7691	0,4913	367,2852	0,7569
292,3966	1,2776	292,3346	0,4515	343,7497	0,7025
253,9827	1,1890	253,9155	0,4070	290,7418	0,6607
215,4964	1,1093	215,4964	0,3690	252,5658	0,6129
177,1756	0,9856	177,0670	0,2988	214,3588	0,5709
138,7513	0,8948	138,6376	0,2575	190,9887	0,5446
92,8284	0,7077	92,6319	0,1792	152,9213	0,4759
				107,1018	0,3748

5. References

S1 Walczak, A.; Stefankiewicz, A. R., pH-Induced Linkage Isomerism of Pd(II) Complexes: A Pathway to Air- and Water-Stable Suzuki–Miyaura-Reaction Catalysts. *Inorg. Chem.* **2018**, *57*, 471-477.

S2 Abdine, R. A. A.; Kurpik, G.; Walczak, A.; Aeash, S. A. A.; Stefankiewicz, A. R.; Monnier, F.; Taillefer, M., Mild temperature amination of aryl iodides and aryl

bromides with aqueous ammonia in the presence of CuBr and pyridyldiketone ligands**.** *J. Catal.* **2019**, *376*, 119-122.
